# Increasing support for the next generation of clinical trials leaders

**DOI:** 10.1186/s40814-016-0073-z

**Published:** 2016-07-18

**Authors:** Peter Thompson, James Fenton, Lisa Cotterill, James P. Neilson

**Affiliations:** 1 NIHR Trainees Coordinating Centre, Leeds Innovation Centre, 103 Clarendon Road, Leeds, UK; 2 Department of Women’s and Children’s Health, The University of Liverpool, Liverpool, UK

**Keywords:** Clinical trials training, NIHR, Fellowships

## Abstract

The National Institute for Health Research (NIHR) has identified a gap in the number of people it funds who are on a pathway to become future leaders of clinical trials, compared to how much the NIHR invests in clinical trials. In order to support the clinical trials of tomorrow, it is vital that the right people are supported now to lead these trials. To address this issue, NIHR organised a workshop with key stakeholders to understand the barriers to embarking on a clinical trials career and explore initiatives to increase capacity and capability in clinical trials. The output from the workshop was a set of recommendations which NIHR is now considering to shape future support.

## Main text

### Background

The National Institute for Health Research (NIHR) is the research arm of the National Health Service (NHS) in England. It is funded by the Department of Health in order to deliver the Government’s strategy for applied health research (http://www.nihr.ac.uk/about/). The vision of the NIHR is, ‘To improve the health and wealth of the nation through research’, and one of its aims in order to realise this vision is to ‘Attract, develop and retain the best research professionals to conduct people-based research’. One of the main ways in which NIHR does this is through its research training programmes which are designed to create the applied health research leaders of the future (www.nihr.ac.uk/funding/training-programmes.htm). The NIHR does not provide training (i.e. Masters courses, short courses) through these programmes; rather, it provides funding and support to allow the best people to undertake excellent research and extensive training and development programmes based within high-calibre organisations. There are funding opportunities available for all professions, clinical and non-clinical, from masters to professorial level. These opportunities allow people to undertake research training through doing research across a range of disciplines and research methodologies. Training in clinical trials represents an important area in which people undertake training as part of an NIHR research training award. Throughout this letter where training in clinical trials is mentioned, this refers to the training undertaken as part of a NIHR research training award, not training provided directly by NIHR. Details of the guidance currently given to applicants for NIHR research training awards can be found within the latest application guidance notes (e.g. http://www.nihr.ac.uk/documents/funding/Training-Programmes/TCC-Fellowships-Guidance-Notes-2016.pdf).

Applicants to the NIHR research training programmes often include a clinical trial, feasibility study or pilot study, or other clinical trials training elements as part of their proposed research and/or training and development programme. Applicants are encouraged to work with a Clinical Trials Unit (CTU) where appropriate and must have the right level of trials experience in their supervisory team. Applicants are also encouraged to think about the scope of any trial or feasibility study in relation to their research training award to ensure that it is realistic to complete within the award timescale and whether it represents a good training vehicle. However, analysis of the NIHR training portfolio, by looking at the type of research proposed within research training award applications, has identified a gap in the number of people undertaking trials training, with approximately 20 % of funded fellowships including a trial or feasibility study. This compares to 59 % of completed Research for Patient Benefit (RfPB) grants being feasibility studies or randomised controlled trials (RCTs) as of October 2013. The RfPB programme represents a first opportunity for many NIHR trainees to gain experience of applying for and being awarded a significant research grant, and whilst it is not necessarily expected that the profile of research training awards in terms of research areas (e.g. UKCRC Health Research Categories) and methodologies (e.g. systematic review, feasibility study, cohort study) should exactly mirror that of the NIHR research programmes, it is NIHR’s view that they should broadly align. This will ensure that the profile of new researchers who will become the research leaders of tomorrow are able to match the requirements, in terms of skills and expertise, needed to deliver future NIHR research. Considering the amount of investment by NIHR in RCTs (774 active RCTs in May 2014 with a research cost of £880 million), it is very important that NIHR trains the individuals capable of leading trials in the future. To help understand barriers to individuals undertaking trials training and to explore initiatives to increase capacity and capability in clinical trials amongst trainees, NIHR set up a workshop with key stakeholders which was held on 29 June 2015. Details of the participants are given in Table 1 and were selected to provide broad representation of the various interested parties from NIHR, Department of Health, Clinical Trials Units and other funders.

**Table 1:** Names and affiliations of attendees at the NIHR Clinical Trials Training workshop

Workshop attendees	
Professor Jim Neilson—Dean for NIHR Faculty Trainees (Chair)	
Professor Dave Jones—Dean for NIHR Faculty Trainees elect	
Dr Lisa Cotterill—Director, NIHR Trainees Coordinating Centre	
Dr Anthony Gordon—NIHR Clinician Scientist	
Dr Carsten Flohr—NIHR Career Development Fellow	
Professor Chris Hatton—Co-Director, RDS North West	
Professor David Armstrong—RfPB Programme Director	
Professor Deborah Ashby—Co-Director, Imperial Clinical Trials Unit	
Professor Elaine McColl—Director, Newcastle Clinical Trials Unit	
Dr Gillian Lancaster—Editor in Chief, Pilot and Feasibility Studies	
Professor Hywel Williams—Chair, HTA Commissioning Board and HTA Deputy Programme Director	
Professor Ian Russell—Emeritus Professor of Clinical Trials	
Professor Janet Peacock—Professor of Medical Statistics	
Professor Jenny Hewison—Chair, HTA Monitoring Strategy Group and former Chair, NIHR Career Development and Senior Research Fellowships Panel	
Professor Julia Brown—Director, Leeds Institute of Clinical Trials Research and Chair, NIHR Doctoral Research Fellowships Panel	
Ms Julie Bishop—Policy Manager, Sponsorship, Performance and Workforce, Department of Health	
Dr Liz Tremain, Senior Programme Manager, NIHR Trials Overview, NETSCC	
Professor Steve Smye, Theme Director, NIHR Clinical Research Network	
Professor Tony Marson, Deputy Director, MRC North West Hub for Trials Methodology Research	
Dr Peter Thompson, Assistant Director, Personal Awards, NIHR TCC	
Dr James Fenton, Assistant Director, Institutional Awards, NIHR TCC	
Mr Tom Pratt, Programme Manager, Clinical Trials Fellowships, NIHR TCC	

### Workshop discussion

The aims of the workshop were to:

- Share the current provision for trainees interested in a career as a clinical trialist
- Understand any barriers to trainees embarking on a career in clinical trials
- Understand any barriers to institutions supporting trainees involved in clinical trials
- Explore initiatives to increase capacity and capability in clinical trials amongst NIHR trainees

The workshop heard views from across NIHR about the current provision for clinical trials training and potential ways forward, including the current provision of research training awards and the viewpoints of a CTU director, a current NIHR trainee, from NIHR’s research programmes and the NIHR Clinical Research Network. The discussion then focussed on how a trial fits into a fellowship and whether this represents a good training vehicle for someone interested in becoming a future trials leader. Training awards which do include a clinical trial primarily focus on feasibility studies and less on full trials. A lot of discussion took place focussed on what a fellowship based around clinical trials training should look like; for example, some successful applicants have used a fellowship to undertake a lot of the groundwork and preparatory research before going onto to gain further funding for a feasibility, pilot study or full trial. The benefit of working with a CTU to gain experience in a wide range of trial activities was also discussed.

The second part of the workshop was structured into breakout groups to discuss the following points:

- Are training programmes fit for purpose?
- What does a career pathway look like?
- How do trainees/researchers move through the NIHR pipeline?

Each of the breakout groups had a facilitator who recorded the key points from each discussion. Each group nominated a spokesperson to feed back to the wider group the key points recorded, which informed a further discussion by the whole group. The meeting Chair facilitated these discussions, and recommendations were agreed by consensus decision-making.

In looking at the training programmes, the workshop concluded that work should be done to investigate providing more flexibility for people wanting to apply for a NIHR Clinical Trials Fellowship (CTF). NIHR CTFs are 6-month fellowships open to already funded NIHR trainees who are interested in undertaking an intense period of clinical trials training in partnership with a CTU following the conclusion of their current NIHR research training award. The workshop also concluded that consideration should be given to expanding the scope of the NIHR Transitional Research Fellowship (TRF) to provide a route into clinical trials for post-doctoral trainees with little trials experience. NIHR TRFs are currently targeted at researchers from a basic science background who want to transition into applied health research and at researchers returning from a significant career break. Undertaking a TRF with focus on moving into clinical trials could then put applicants in a strong position to apply for further trials funding or fellowships, to lead on a small feasibility study, for example. To ensure that training takes place within a high-quality environment, encouragement, or a requirement, to link with a CTU could be given. The possibility of providing fellowships with explicit links to already funded trials was also discussed.

In thinking about career pathways, the workshop considered only those training to become future Chief Investigators rather than the myriad of other roles involved with clinical trials. It was agreed that a clear career pathway for future Chief Investigators does not currently exist and that career pathways for researchers who undertake trials training is currently based more upon their clinical academic pathway (for clinician researchers). Expanding the scope of the TRF as described above could help with this, as would wider dissemination of case studies showcasing people who have forged out a career in clinical trials.

Discussions around moving trainees through the NIHR pathway focussed again on those training to become future Chief Investigators. There was an agreement that training awards should have a focus on feasibility and the wider aspects of clinical trials so that trainees are in a strong position to apply for trials funding in the future from programmes like Health Technology Assessment (HTA). The group also thought that developing some key skills for those training in clinical trials would be helpful. This could help, for example, someone wanting to put together a PhD programme with a focus on trials training.

### Outcomes

The outcome of the workshop was a set of recommendations that have been developed into a specific set of proposals through a task and finish group which NIHR set up following the workshop. These proposals are now under consideration by NIHR, and any changes to research training programmes as a result of these proposals will be announced during course of 2016.

The original aims of the workshop had been to:

- Share the current provision for trainees interested in a career as a clinical trialist
- Understand any barriers to trainees embarking on a career in clinical trials
- Understand any barriers to institutions supporting trainees involved in clinical trials
- Explore initiatives to increase capacity and capability in clinical trials amongst NIHR trainees

The workshop represented a good opportunity to present the current provision of research training opportunities provided by NIHR which allow for training in clinical trials, and as a result of the discussions, several potential barriers to people taking up these opportunities emerged. These are summarised below:

- The timescale of a clinical trial does not necessarily fit with that for a personal research training award.
- The additional staff and funds required to run a large trial do not necessarily fit within the scope of what can be provided by a personal research training award.
- The additional time constraints associated with registering for a PhD are not always compatible with the timescales of a clinical trial.
- Undertaking a clinical trial or research on a trials-related topic may not be as attractive to potential PhD students as more traditional lab-based research.
- Applicants are not necessarily aware of how current NIHR awards can be utilised to further skills and experience in clinical trials.
- There is not a clear career pathway for researchers looking to become future Chief Investigators.
- Additional flexibility may be required in some schemes, particularly the CTF, to increase their attractiveness to potential applicants.

The identification of potential barriers helped shape the recommendations which came out of the workshop, all of which were agreed upon with the aim of overcoming these barriers and increasing capacity and capability in clinical trials amongst NIHR trainees.

These recommendations are broadly summarised below:

- Increase flexibility and availability of training programmes for clinical trials training
- Increase dissemination of opportunities for clinical trials training
- Explore linking trials training into the broader research pathway
- Consider key skills for different stages of clinical trials training
- Consider how research training awards can best prepare trainees for a career as a trials leader when developing recommendations into concrete proposals

The task and finish group which developed these recommendations further was made up of a sub-section of the attendees at the workshop reported here and worked to the following terms of reference:

1. To develop proposals based upon the recommendations of the NIHR Clinical Trials Training workshop for future clinical trials training within NIHR
2. To advise on the implementation of these proposals within the current structure of NIHR research training programmes

Whilst there are no plans to repeat the workshop reported here, any changes that are implemented as a result of this workshop will be reviewed in the future and where appropriate, and if required, further workshops may be organised.

## Abbreviations

CTF, Clinical Trials Fellowship; CTU, Clinical Trials Unit; HTA, Health Technology Assessment; NHS, National Health Service; NIHR, National Institute for Health Research; RCT, randomised controlled trial; RfPB, Research for Patient Benefit; TRF, Transitional Research Fellowship

